# The cytokine environment influence on human skin–derived T cells

**DOI:** 10.1096/fj.201801416R

**Published:** 2019-02-26

**Authors:** Philip Kienzl, Romana Polacek, Manuel Reithofer, René Reitermaier, Pia Hagenbach, Pooja Tajpara, Martin Vierhapper, Maria Gschwandtner, Michael Mildner, Beatrice Jahn-Schmid, Adelheid Elbe-Bürger

**Affiliations:** * Department of Dermatology, Medical University of Vienna, Vienna, Austria;; † Institute of Pathophysiology and Allergy Research, Medical University of Vienna, Vienna, Austria;; ‡ Division of Plastic and Reconstructive Surgery, Department of Surgery, Medical University of Vienna, Vienna, Austria;; § Research Division of Biology and Pathobiology of the Skin, Department of Dermatology, Medical University of Vienna, Vienna, Austria

**Keywords:** T-cell plasticity, IL-9, neutrophil, IL-8, CXCL13

## Abstract

Skin resident T cells provide immediate immunologic responses at their specific location and play a role in the pathogenesis of skin diseases such as psoriasis. Recently, IL-9–producing T cells were described as a major T-cell subtype present in the skin, but knowledge on the biology and *in situ* regulation of this T-cell subtype is scarce. Here, we investigated the cytokine influence on skin T cells with focus on IL-9–producing T cells because a better understanding of their biology may identify novel therapeutic approaches. Healthy human skin biopsies were cultured either in the presence of IL-2, IL-4, and TGF-β [T helper (T_h_)9–promoting condition (T_h_9-PC)] or IL-2 and IL-15 [standard condition (SC)]. Paired analysis of enzymatically isolated skin T cells and emigrated T cells after 4 wk of skin culture showed significant alterations of T-cell phenotypes, cytokine production, and IL-9–producing T-cell frequency. RNA sequencing analysis revealed differentially regulated pathways and identified *CXCL8* and *CXCL13* as top up-regulated genes in T_h_9-PC compared with SC. Functionally supernatant of stimulated skin-derived T cells, CXCL8 and CXCL13 increased neutrophil survival. We report that the cytokine environment alters skin-derived T-cell phenotype and functional properties.—Kienzl, P., Polacek, R., Reithofer, M., Reitermaier, R., Hagenbach, P., Tajpara, P., Vierhapper, M., Gschwandtner, M., Mildner, M. Jahn-Schmid, B., Elbe-Bürger, A. The cytokine environment influence on human skin–derived T cells.

Human skin not only forms a physical barrier to the outside environment but also harbors a multitude of cells of the innate and adaptive immune system, thereby constituting the immunologic barrier (1–4). Clark *et al.* (5) estimated that ∼20 billion resident T cells are present in the healthy skin of an average individual, which react locally and rapidly to invading pathogens. A major role for their retainment in the skin is attributed to the early activation marker CD69 that is able to negatively regulate the migration molecule sphingosine-1-phosphate receptor 1, and thereby prevents T cells from leaving the skin (6–8). CD103 was described as another skin homing marker (9, 10). Cutaneous lymphocyte-associated antigen (CLA) allows T cells to tether to blood vessel walls, enabling them to exit the bloodstream and migrate into skin (11, 12), and C-C motif chemokine receptor 4 senses chemokine signals that guide the cell from the bloodstream into skin tissue (13).

T helper (T_h_)9 cells, characterized by the expression of IL-9, have recently been described as the T_h_ cell subset present in the skin apart from regulatory T (T_reg_) cells, T_h_1, T_h_2, and T_h_17 cells and are also found under steady-state conditions (14–16). They are involved in the immune reaction against extracellular pathogens, especially against *Candida albicans*, and mediate antitumor immunity against melanoma in a mouse model (14, 17). In addition, IL-9^+^ T cells are found in psoriatic and atopic dermatitis lesions, suggesting a possible role in inflammatory skin diseases (14) as well as in mycosis fungoides (18).

The combined effects of IL-2, IL-4, and TGF-β on naive T cells promote their differentiation into T_h_9 cells (19, 20). Skin can potentially provide a milieu favoring T_h_9 differentiation because keratinocytes are well-known producers of TGF-β (21–23), and IL-4 is released by activated antigen-presenting cells (3, 21, 24) and type 2 innate lymphoid cells (25). IL-2 is primarily produced by activated CD4^+^ T cells (26) as demonstrated for inflammatory skin diseases (27). IL-2 promotes IL-9 gene expression *via* the signal transducer and activator of transcription (STAT) 5 and regulates the expression of IL-4Rα (19, 28). IL-4 does not impact IL-9 expression directly but rather through the sSTAT6-mediated induction of the IL-9 transcription factor IFN regulatory factor 4 (*IRF4*), among others. *PU.1* (SPI1) was described as an important transcription factor for IL-9 production and is regulated by TGF-β (29, 30). Nonetheless, no master transcription factor as found in other T_h_ cell subsets has been identified yet (20). In addition, to our knowledge, it is currently not possible to isolate viable IL-9–producing T cells because of a lack of specific surface marker proteins.

Here, we studied the influence of the cytokine milieu on emigrating skin-derived T cells and found significant phenotypical and functional alterations. Because most studies on T_h_9 cells were conducted in mice, we aimed to characterize IL-9–producing T cells in healthy human skin in more detail. Skin IL-9–producing T cells exhibited a plastic phenotype dependent on the cytokine milieu but did not coproduce signature cytokines from T_h_1, T_h_2, or T_h_17 cells.

## MATERIALS AND METHODS

Details on experimental procedures are provided in the Supplemental Data.

### Study approval

This study was approved by the Ethics Committee of the Medical University of Vienna. Healthy human skin was obtained from patients undergoing cosmetic surgeries after informed consent was obtained and according to the Declaration of Helsinki. Experiments were performed within the first 24 h after surgical removal.

### Skin T-cell isolation

For freshly isolated (FI) T-cell stimulation experiments, Collagenase P (Roche, Basel, Switzerland) digestion of fresh human skin was used essentially as previously described (31). In brief, dermatome-cut skin specimens were digested in complete Roswell Park Memorial Institute (RPMI) 1640 medium (Thermo Fisher Scientific, Waltham, MA, USA) supplemented with 10% fetal bovine serum and 1% penicillin-streptomycin (complete RPMI) containing 1 mg/ml Collagenase P (Roche) at 37°C on a rotator for 3 h. Next, 200 U/ml DNase I (Roche) were added and incubated for 15 min at 37°C. The cell suspension was diluted with 10 mM EDTA/PBS and filtered through 100- and 40-µm cell strainers. Dead cells were eliminated using a Dead Cell Removal Kit (Miltenyi Biotec, Bergisch Gladbach, Germany), and T cells were enriched (purity range: 91–98%) with CD2 microbeads (Miltenyi Biotec) according to manufacturer’s instructions. Next, either IL-9 staining was performed or cells were further stimulated. For flow-cytometric analysis of surface proteins on FI skin T cells, the Whole Skin Dissociation Kit (Miltenyi Biotec) was used according to manufacturer’s instructions to digest human skin.

### Skin explant cultures

Skin explant cultures were prepared as previously described in Clark *et al.* (5) with modifications. Briefly, skin punch biopsies (5 mm) were minced, subsequently loaded onto collagen G (Biochrom, Berlin, Germany)–coated tantalum cell foam matrix grids (Ultramet, Pacoima, CA, USA), and placed in 24-well plates submerged in complete RPMI 1640, either with 100 U/ml IL-2, 2 ng/ml IL-4, and 10 ng/ml TGF-β or 100 U/ml IL-2 and 10 ng/ml IL-15 (Peprotech, Rocky Hill, NJ, USA). The IL-4 and TGF-β concentrations for maximal differentiation and expansion of T_h_9 cells in our skin explant model had to be evaluated in a set of experiments (unpublished results) because, to our knowledge, there are no publications utilizing this specific cytokine combination in this setting in humans. Cells were harvested after 4 wk of culture and either analyzed by flow cytometry or further stimulated.

### T-cell stimulation

Cells were seeded in round-bottom 96-well plates at a density of 5 × 10^5^/ml in complete RPMI 1640 medium containing 50 µM 2-mercaptoethanol (Thermo Fisher Scientific) and Dynabeads Human T-Activator CD3/CD28 (bead-to-cell ratio of 1:1; Thermo Fisher Scientific). After 3 d of culture, beads were magnetically removed and the cells incubated in complete RPMI 1640 medium supplemented with 1× cell-stimulation cocktail (CSC; Thermo Fisher Scientific) plus transport inhibitors for 4 h. After harvesting, an aliquot was stained for flow cytometry, and the other cells were layered on Ficoll Paque Plus (GE Healthcare, Waukesha, WI, USA) for density gradient centrifugation to remove dead cells and debris. Finally, adhesion slides, RNA, and protein lysates were prepared.

### Immunofluorescence staining

Antibodies are specified in Supplemental Table S1. Species and isotype-specific secondary antibodies αrabbit Alexa Fluor 488 and αmouse (H+L) Alexa Fluor 546 (Thermo Fisher Scientific) were used. Images were acquired using an AX70 Microscope (Olympus, Tokyo, Japan) with the imaging software MetaMorph (Molecular Devices, Sunnyvale, CA, USA) or an LSM 500 Microscope (Carl Zeiss, Oberkochen, Germany) equipped with Zen software (Carl Zeiss).

### Flow cytometry

Fluorochrome-labeled antibodies are listed in Supplemental Table S1. Samples were acquired on a BD FacsVerse Flow Cytometer (BD Biosciences, San Jose, CA, USA) with FACSuite v.1.0.5.3841 software (BD Biosciences) and analyzed using FlowJo v.10.0.7 (BD Biosciences).

### Analysis of cytokine concentrations

Supernatants from skin explant cultures after 4 wk and after αCD3 and αCD28 bead stimulation of emigrated skin T cells after 4 wk of explant culture were collected for bead array analysis with LegendPlex Human T_h_ Cytokine Panel (13-plex; BioLegend, San Diego, CA, USA). The assay was performed according to the manufacturer’s instructions. Cytokine concentrations were calculated using the LegendPlex v.7.0 software tool (BioLegend).

### Western blot

FI, bead-stimulated, and CSC-restimulated skin T cells were lysed in SDS-PAGE loading buffer, sonicated, and denatured with 0.1 M DL-DTT (MilliporeSigma).

### Transcriptome analyses

E.Z.N.A. MicroElute Total RNA Kit (Omega Bio-tek, Norcross, GA, USA) with DNase I On-Column Digestion was used for the isolation of total RNA according to the manufacturer’s instructions. Sequencing libraries were prepared (Core Facility Genomics, Medical University of Vienna) using the NebNext Ultra Directional RNA Library Prep Kit for Illumina according to the manufacturer’s protocols (New England Biolabs, Ipswich, MA, USA).

### Isolation of peripheral blood mononuclear cells and culture

**Figure 1:**
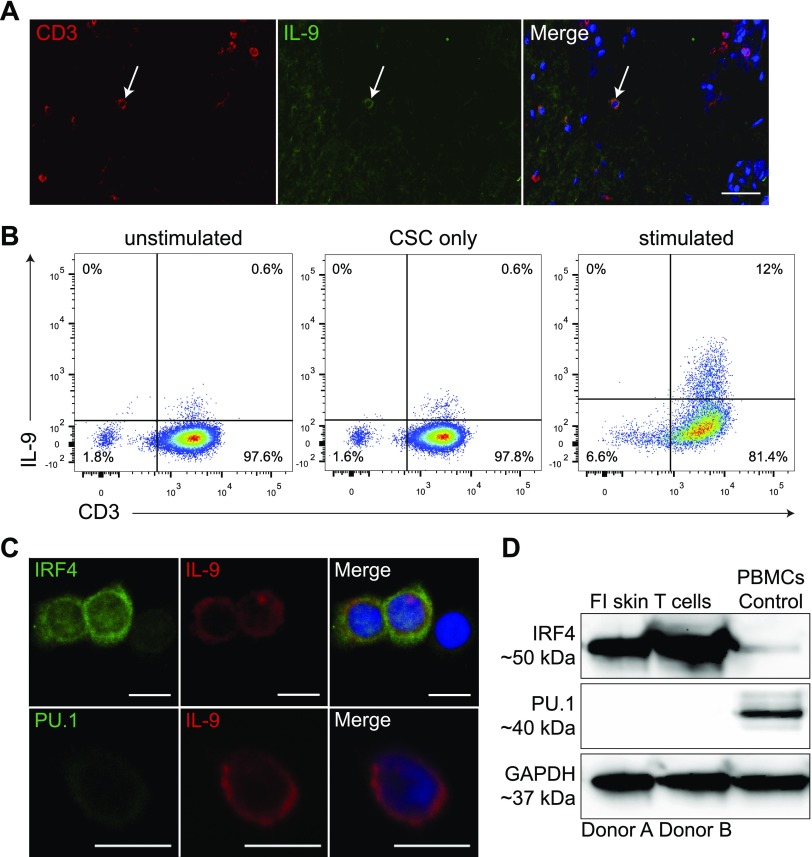
IL-9–producing T cells in healthy human skin express IRF4. *A*) Representative 2-color immunofluorescence staining of CD3 and IL-9 in cryosections of human skin (*n* = 3). White arrow marks an IL-9^+^ T cell within the lower dermal compartment. Scale bar, 50 µm. *B*) Flow cytometric analysis of IL-9^+^ T cells in FI unstimulated (left), FI CSC-stimulated (middle), and 3-d αCD3- and αCD28-stimulated and CSC-restimulated skin-derived T cells (right). Quadrants were set according to isotype-matched control staining. *C*) Two-color immunofluorescence staining for IRF4 and IL-9 (upper panel) and PU.1 and IL-9 (lower panel). FI skin T cells were αCD3 and αCD28 bead–stimulated and CSC-activated. Shown is one representative experiment of 3 independent donors. Scale bar, 10 µm. *D*) Protein expression of indicated markers from 3-d αCD3 and αCD28 bead–stimulated and CSC-restimulated FI skin T cells from 2 representative donors was analyzed by Western blot, and 2 representative donors of 5 are shown. PBMC lysate was included as control.

Living peripheral blood mononuclear cells (PBMCs) and protein lysates thereof (Fig. 1*D*) were prepared essentially as previously described in Gschwandtner *et al.* (32). Cells were cultured in serum-free TexMacs (Miltenyi Biotec) medium alone or with chemokine (C-X-C motif) ligand 8 (100 ng/ml), CXCL13 (100 ng/ml), or both cytokines combined in round-bottom 96-well plates at 37°C, 5% CO_2_, and 95% humidity for 48 h. Dead cells were excluded with fixable viability dye eFluor 450 (Thermo Fisher Scientific). The following antibodies were used for flow cytometry staining: CD3-PE, CD4-Brilliant Violet 510, CD8-allophycocyanin (APC)-Vio770, CLA-FITC, CCR4-APC (clone 205410), CD69-PE-Cy7, and CD103-PerCP-eFluor710 (Supplemental Table S1). Analysis was carried using BD FACSVerse.

### Migration assay

PBMCs and neutrophils (purity >95%) were isolated as described in Gschwandtner *et al.* and Polak *et al.* (32, 33), and 2 × 10^5^ cells in 30 μl RPMI 1640 medium were added onto the membrane of the migration system (ChemoTx; Neuro Probe, Gaithersburg, MD, USA). The lower compartment was filled with 300 μl of 100 ng/ml CXCL8 (neutrophils) or CXCL13 (PBMCs). After 2 h, cells were harvested and counted using BD FACSVerse.

### Cytokine stimulation of neutrophils and human myeloperoxidase ELISA

Isolated neutrophils were cultured in 200 μl RPMI 1640 medium at a cell density of 10^3^/μl containing 11% supernatant of stimulated T cells derived from 4-wk skin explant cultures or complete RPMI 1640 medium as control (Supplemental Fig. S3*D*, *E*) or in RPMI 1640 medium containing 100 ng/ml CXCL8 or CXCL13 for 24 h. Before and after culture, viability of neutrophils was assessed using Annexin V and 7-aminoactinomycin D (7-AAD) staining. For this, cells were first incubated with Annexin V Alexa Fluor 488 antibody (Thermo Fisher Scientific) in Annexin V Binding Buffer (BioLegend) for 15 min, and then 7-AAD (BD Biosciences) was added before analysis by flow cytometry.

Human myeloperoxidase (MPO) concentrations in cell and skin culture supernatants were determined by the Human MPO DuoSet ELISA Kit (R&D Systems, Minneapolis, MN, USA) according to the manufacturer’s instructions. Absorbance was measured using a Multiskan photometer (Thermo Fisher Scientific) at 450 nm, and background was subtracted before fitting the values to a 4-parametric logistic model of the standards.

### Statistical analysis

Graphical illustrations and statistical computations were performed using Prism 6 (GraphPad Software, La Jolla, CA, USA). Values of *P* < 0.05 were regarded as significant. The designation *n* in figure legends generally refers to biologic replicates, except when stated otherwise.

## RESULTS

### The transcription factor IRF4, but not PU.1, is expressed in dermal IL-9–producing T cells

Two-color immunofluorescence staining on healthy human skin cryosections revealed that most of the CD3^+^IL-9^+^ T cells were located in the dermis but never in the epidermis (**Fig. 1*A*** and Supplemental Fig. S1*A*), confirming previously published data in Schlapbach *et al.* (14). In addition, CD3^−^IL-9^+^ cells were found within the dermis and most likely represent mast cells or innate lymphoid cells, which are known to secrete IL-9 (34–38). Seventeen percent of αCD3 and αCD28 bead–stimulated and CSC-restimulated FI dermal T cells were positive for IL-9, whereas only very few unstimulated or CSC-stimulated dermal T cells were IL-9^+^ (Fig. 1*B*). IRF4, but not PU.1, was identified in the cytoplasm and nucleus in IL-9^+^ T cells (Fig. 1*C*). The staining patterns of IRF4 and IL-9 are similar to previously published results in thymic cells and skin T cells, respectively (14, 39). Immunofluorescence data were confirmed by qualitative Western blot analysis with identical results (Fig. 1*D*).

### Cytokine environment influences the phenotype of human skin–derived T cells

**Figure 2:**
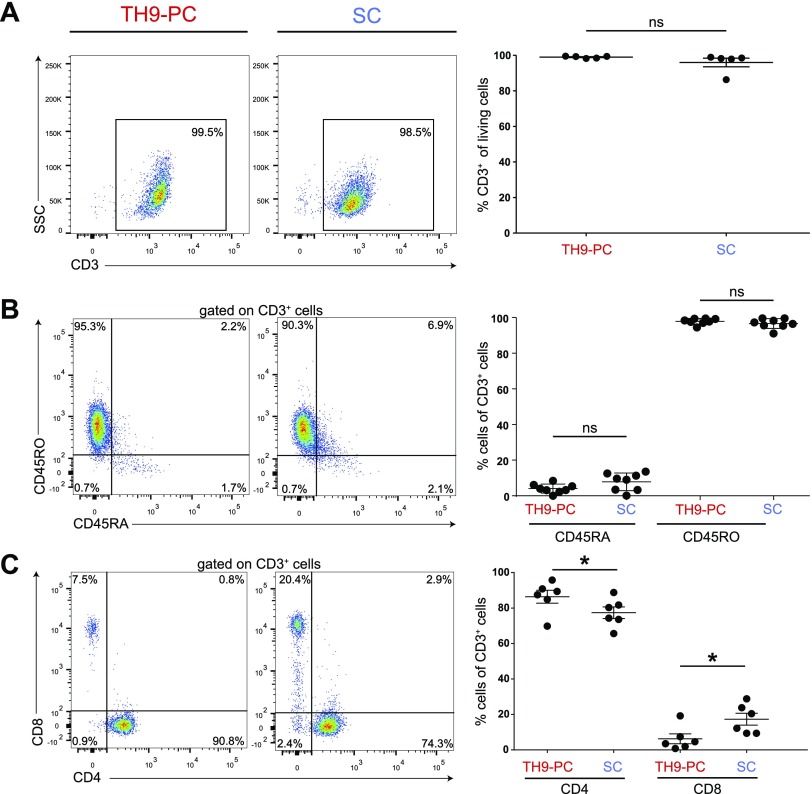
Phenotypic profile of T cells harvested from skin explant cultures after 4 wk. Representative flow cytometry dot plots (left) and quantification statistics (right; *n* = 5–8) of the indicated surface markers on cells cultured under T_h_9-PC or SC (SSC/CD3, *A*; CD45RO/CD45RA, *B*; CD8/DC4, *C*, IL-9/CD3, *D*; CCR4/CLA, *E*; CD69/CD103, *F*). Cells (*D*) were αCD3 and αCD28 bead–stimulated and CSC-restimulated 3 d. Quadrants were set according to isotype-matched control staining. Ns, not significant. Means ± sem are shown, and data were analyzed using Wilcoxon’s rank-sum test. *P ≤ 0.05.

Next, we investigated the cytokine influence on human skin–derived T cells and the potential to propagate IL-9–producing T cells in an adapted *ex vivo* skin culture model to learn more about their biology (40). In the presence of either T_h_9-promoting conditions (T_h_9-PC; IL-2, IL-4, and TGF-β) or standard conditions (SCs; IL-2 and IL-15), the number of cells emigrated per well steadily increased. Approximately 15% of the cells, irrespective of the cytokines provided, were Ki-67^+^ after 4 wk, suggesting that proliferation contributes to overall cell numbers (Supplemental Fig. S1*B*, *C*). Emigrated cells from T_h_9-PC were exclusively CD45RO^+^CD45RA^−^CD3^+^ memory T cells, whereas from SC (besides the overwhelming majority of CD45RO^+^CD45RA^−^CD3^+^ T cells) (**Fig. 2*A, B***), a small CD3^−^ population (range: 1.7–16.0%) was consistently observed and consisted mainly of CD56^+^ natural killer (NK) cells (Supplemental Fig. S1*D*). Under both conditions, we repeatedly found more CD4^+^ T cells than CD8^+^ T cells (Fig. 2*C*). Equal numbers of cells expressed the T_reg_ cell master transcription factor forkhead box P3 under both culture conditions (Supplemental Fig. S1*E*). However, T_reg_-differentiating cytokines (IL-2 and TGF-β) increased FoxP3^+^ T-cell densities significantly (Supplemental Fig. S1*E*). As hypothesized, significantly more IL-9^+^ T cells were found in T_h_9-PC as compared with SC (Fig. 2*D*). Frequencies of CLA^+^ and CD103^+^ T cells were unchanged under both culture conditions. In contrast, CCR4^+^ and CD69^+^ T-cell frequencies were extremely low under SC and T_h_9-PC, respectively (Fig. 2*E, F*). In accordance with our flow cytometry data (Fig. 2*D*), a significantly higher IL-9 concentration was observed in T_h_9-PC compared with SC when supernatants were analyzed after 4 wk of culture. The T_h_2-associated cytokine IL-13 was significantly higher under SC, and IFN-γ levels were below detection limit under T_h_9-PC (Supplemental Fig. S1*F*).

### Culture conditions change skin homing–marker expression and are crucial for maintaining the IL-9–expressing phenotype

**Figure 3:**
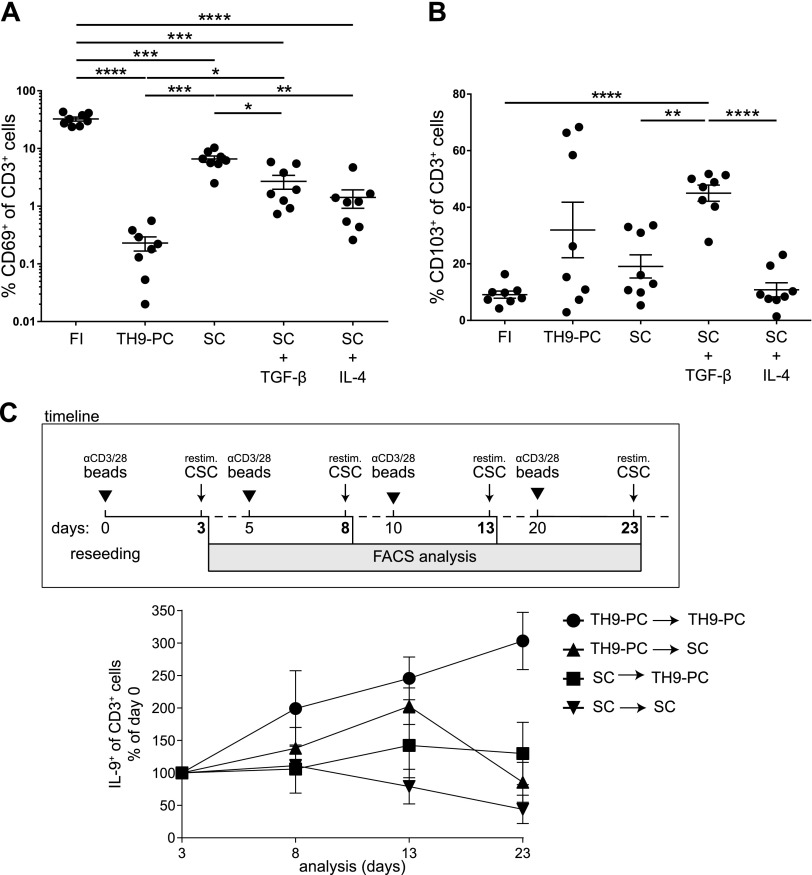
Culture conditions alter CD69 and CD103 expression on skin T cells. *A*, *B*) Percentage of CD69 and CD103 cells among skin T cells (FI) or harvested after 4 wk from skin explant cultures under indicated conditions. Paired data are means ± sem and were analyzed using repeated measures with 1-way ANOVA (Holm-Sidak *post hoc*). *P ≤ 0.05, **P ≤ 0.01, ***P ≤ 0.001, ****P ≤ 0.0001. Only significant differences are indicated. *C*) T cells from 4-wk cultures were reseeded (d 0; 10^5^ T cells/well) and stimulated as well as restimulated according to the scheme. Percentage of IL-9^+^ T cells upon reseeding T cells under indicated conditions. Before each analysis, cells were αCD3 and αCD28 bead–stimulated and CSC-restimulated 3 d at the indicated time points (*n* = 3). Data are means ± sem.

To compare the skin homing–marker profile on T cells before and after culture from the same donors (paired data), T cells were isolated from whole-skin specimens directly (FI) and harvested after 4 wk of skin explant culture. FI T cells expressed very high levels of CD69, whereas a significantly lower frequency of CD69^+^ T cells was observed under both culture conditions, especially under T_h_9-PC (**Fig. 3*A***). To identify the responsible cytokine, cells from FI, T_h_9-PC, SC, SC plus TGF-β, or SC plus IL-4 were analyzed. Both cytokines significantly diminished the frequency of CD69^+^ T cells, but the stronger effect was exerted by IL-4 (Fig. 3*A*). Analysis of FI T cells revealed a low frequency of CD103^+^ T cells. Surprisingly, their density increased upon skin explant culture and was higher under T_h_9-PC as compared with SC when using the same culture setup as described for CD69. Here, the effect was mediated by TGF-β but not by IL-4 (Fig. 3*B*). Applying the same experimental strategy, we studied CCR4 expression on skin T cells and changes upon the addition of either TGF-β or IL-4. Neither TGF-β nor IL-4 restored the CCR4^+^ T-cell frequency when compared with the T_h_9-PC group, suggesting an additive effect of TGF-β and IL-4 on CCR4 expression (Supplemental Fig. S3*A*).

To test the stability of the IL-9–producing T-cell phenotype, T cells from both culture conditions were reseeded after 4 wk either in their previous cytokine milieu or in the respective other cytokine condition (Fig. 3*C*, scheme). As expected, the IL-9^+^ T-cell frequency steadily increased under continuous T_h_9-PC, whereas it initially slightly increased and then remained constant when cells were reseeded from SC to T_h_9-PC. Intriguingly, the IL-9^+^ T-cell frequency among T cells that were previously cultured under T_h_9-PC and further cultured under SC increased up to d 10 and later decreased (Fig. 3*C*). Taken together, these data suggest a cytokine influence on skin homing–marker expression and a dependence of IL-9 expression of T cells on the cytokine environment.

### The vast majority of IL-9–producing T cells do not coexpress T_h_1, T_h_2, or T_h_17 signature cytokines

**Figure 4:**
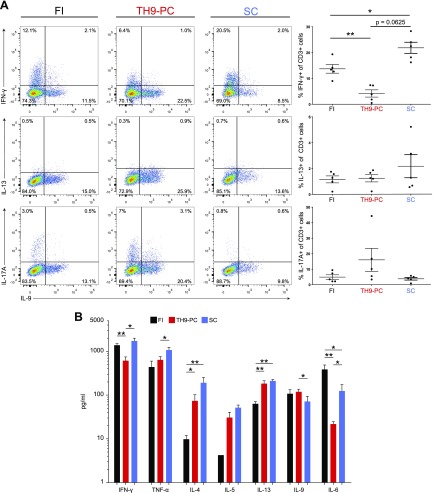
IL-9^+^ skin-derived T cells do not coexpress T_h_1, T_h_2, or T_h_17 signature cytokines. *A*) Flow cytometric dot plots with indicated cytokine analyses and gating on CD3 (left panel) after cells were stimulated with αCD3 and αCD28 beads for 3 d and restimulated with CSC for 4 h. Quadrants were set according to isotype-matched control staining. Percentage of cytokine-expressing T cells among total CD3^+^ T cells was determined from 5 different donors (right panel). *B*) Cytokine concentrations in supernatants from αCD3 and αCD28 bead–stimulated cultures 3 d were determined by cytokine bead array (*n* = 7/group). In the IL-5 group with FI cells, cytokine concentrations of only 2 donors were above the detection limit of the bead array. Data represent means ± sem. FI *vs.* T_h_9-PC and SC were compared using a Mann-Whitney *U* test and T_h_9-PC *vs.* SC using Wilcoxon’s rank-sum test. *P ≤ 0.05, **P ≤ 0.01.

IL-9 was originally described as T_h_2 cytokine because it can be coproduced together with the classic T_h_2 cytokines. Furthermore, T_h_17 cells can express IL-9. However, *bona fide* T_h_9 cells are considered to solely produce IL-9. The characterization of the cytokine profile of FI as well as emigrated T cells from skin explants after 4 wk of culture and after αCD3 and αCD28 and CSC stimulation, irrespective of the culture conditions, revealed that IL-9–producing T cells did not generally coexpress cytokines of other T_h_ cell subsets but represented a distinct T-cell subset (**Fig. 4*A***). Among multiple T_h_-type cytokines tested in supernatants of αCD3 and αCD28 bead–stimulated FI and emigrated skin T cells, IFN-γ and TNF-α concentrations were highest. IL-4, IL-5, and IL-13 were extremely low in FI T-cell supernatants and low in supernatants of emigrated T cells, irrespective of the culture conditions. In contrast, IL-9 levels were significantly higher under T_h_9-PC compared with SC. IL-6 was significantly lower in supernatants of cultured T cells and lowest under T_h_9-PC compared with FI T cells (Fig. 4*B*). Taken together, IL-9–producing T cells do not coexpress other important T_h_-type cytokines. Furthermore, FI and emigrated skin T cells have a differential cytokine expression profile.

### The cytokine environment influences the transcriptional profile of emigrated T cells

**Figure 5:**
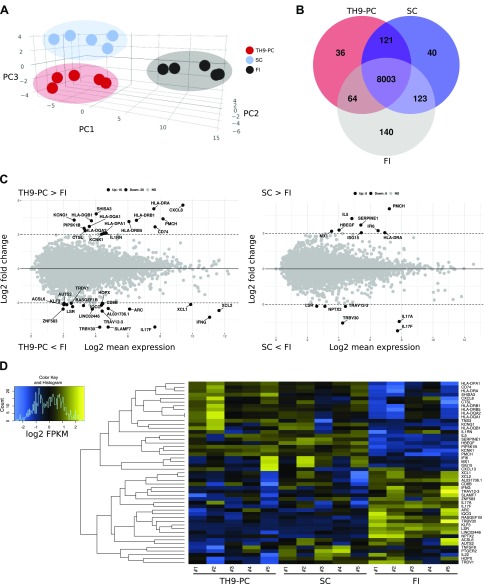
Culture conditions affect the transcriptional profile of skin T cells. RNA-seq analysis of T_h_9-PC, SC, and FI T cells of 5 healthy individuals. *A*) Principal component analysis of the 200 most significantly differentially regulated genes. Color-coded dots represent individual donors from indicated conditions. Principal components 1 and 2 accounted for 83.1% of the total variation. Color-coded ellipses mark clusters. *B*) Venn diagram of expressed genes [mean fragments/kb of transcript/10^6^ (FPKM) >1] among each group. *C*) M (log ratio)/A (mean average)-plot (log_2_ fold change against log_2_ mean expression) showing differential gene expression between indicated conditions. Significantly regulated genes with more than 8-fold change were labeled with their respective gene symbol. *D*) Heatmap illustrating gene expression of differentially regulated genes selected from MA-plot analyses in 5 individuals per condition. Rows represent clustered genes and columns represent donors of each condition.

RNA-sequencing (RNA-seq) analysis was performed, and principal component analysis revealed distinct populations of FI, T_h_9-PC, and SC (**Fig. 5*A***). Ninety-three percent of detected genes were present in T cells in all analyzed conditions (Fig. 5*B*). The *CXCL8* (IL-8) gene as well as genes associated with antigen presentation, such as human leukocyte antigen (*HLA*)-*DP*, *HLA-DQ*, and *HLA-DR*, were up-regulated, whereas *IFN-γ*, *IL-17F,* *CD8*, and variants of T-cell receptor α, β, and δ chains were down-regulated genes in T_h_9-PC compared with FI T cells (Fig. 5*C, D*, and Supplemental Table S2). Genes involved in lymphocyte-mediated immunity, leukocyte activation, and antigen binding were shared between T_h_9-PC and FI T cells. In addition, functional annotation analysis showed enrichment in gene sets associated with cell-cell adhesion (Supplemental Fig. S2*D*). Conversely, genes associated with antiviral response (*MX1*, *ISG15*, and *IFI6*) were higher in SC than in FI (Fig. 5*D* and Supplemental Fig. S2*D*). *CXCL8* and *CXCL13* ranged among the most up-regulated genes, whereas *IL-22* and *IFN-γ* were down-regulated genes in T_h_9-PC compared with SC T cells (Supplemental Fig. S2*A*). IFN-γ was similarly regulated on the protein level (Fig. 4*A*). *IL-17A* and *IL-17F* were significantly down-regulated in SC compared with FI (Fig. 5*C*), a trend that was also seen on the protein level (Fig. 4*A*). Determination of *HLA-DRB1* expression levels on T cells by flow cytometry confirmed a higher HLA-DR–positive T-cell frequency in T_h_9-PC than in SC (Supplemental Fig. S2*B*). Although *IRF4* transcripts were identified by RNA-seq, *PU.1* transcripts were undetectable (Supplemental Table S2), which is in line with our data on the protein level (Fig. 1*D, E*). Importantly, *IL-9* mRNA was significantly higher in T_h_9-PC than in SC (*P* = 0.03) (Supplemental Fig. S2*C*). Our data indicate that the cytokine milieu influences the transcriptional profile of skin T cells.

To investigate the potential involvement of our top hits *CXCL8* and *CXCL13* in skin homing of T cells, we treated PBMCs isolated from human peripheral blood with either cytokine alone or a combination of both. After 48 h of culture, we determined the percentage of CLA^+^, CCR4^+^, CD69^+^, and CD103^+^ T cells using flow cytometry. No significant differences were observed between the tested groups (Supplemental Fig. S3*B*). As a positive control experiment, we performed a migration assay, which showed that significantly more purified neutrophils and PBMCs migrated through the filter membrane when the respective cytokines were present compared with the medium control (Supplemental Fig. S3*C*).

Previous work showed that CXCL8 is an important mediator of neutrophil survival *via* the effects of the serine and threonine protein kinase B (41). To test if the supernatant produced by stimulated T cells derived from 4-wk skin explant cultures affect neutrophil survival, purified neutrophils from human blood were cultured in medium containing supernatant for 24 h. Thereafter, cell survival was assessed by Annexin V and 7-AAD staining. Indeed, we found that the supernatant of stimulated T cells increased the survival of neutrophils significantly when compared with medium control. No significant difference was observed with supernatants from stimulated T cells derived from either T_h_9-PC or SC cultures (Supplemental Fig. S3*D*). MPO is an important enzyme for the generation of reactive oxygen species (42) because it can be produced by neutrophils and kill microbiota (43). When the MPO concentration was determined at the end of the neutrophil culture, we found that compared with the medium control, significantly less MPO was measured in the presence of supernatants derived from stimulated T cells of T_h_9-PC or SC cultures (Supplemental Fig. S3*E*). Unexpectedly, both recombinant CXCL8 and CXCL13 significantly increased neutrophil survival (Supplemental Fig. S3*F*). These data highlight a role of the factors produced by stimulated skin-derived T cells and, in particular, CXCL8 as well as CXCL13 in neutrophil immunobiology.

## DISCUSSION

Over the years, several T-cell subsets have been identified and characterized. Adding more to the complexity is the fact that T cells can be very flexible and, for example, are able to express not just one but many signature cytokines, which is especially evident *in vivo* (44). In this study, we aimed to shed more light on the local regulation of T cells within the skin in terms of the cytokine profile and surface-marker expression in response to cytokine stimulation.

Apart from IFN-γ–producing T cells, here we report that IL-9–producing T cells are a frequent T-cell subtype (mean = 17% of skin T cells) in healthy human skin, thus confirming and extending a previous report (14). *Candida* species are important pathogens that are common in the environment and colonize the skin of up to 70% of healthy humans (45). Unambiguously, it has been shown that T_h_9 cells are specific for *Candida* antigen (14). Because we observed that a substantial number among skin-derived T cells is able to produce IL-9, it is tempting to speculate that IL-9–producing T cells might indeed contribute to controlling *Candida* skin infections. It is conceivable that after infection, IL-9–producing T cells continue to reside locally in the skin. Along this line, RNA-seq identified *CXCL13* and *CXCL8* (*IL-8*), which recruits neutrophils, cells that are able to mediate antifungal defense, to be among the most up-regulated genes in T_h_9-PC compared with SC (46).

*PU.1* was described as one of the major developmental transcription factors for T_h_9 cells and was also present in T cells residing in colon mucosa of ulcerative colitis patients (29, 47, 48). However, we and others (18, 49) could not detect PU.1 in IL-9–producing T cells of healthy human skin. IRF4^+^ T cells were identified in skin from mycosis fungoides lesions and mycosis fungoides cell lines. In allergic contact hypersensitivity, an inflammatory skin disease, PU.1^+^CD3^+^ T cells were found in the epidermis as well as in the dermis. Other transcription factors such as *IRF4* and *IRF8* might regulate the expression of IL-9 in the skin through a different cytokine environment (50). This would argue in favor of skin-resident IL-9–producing T cells being different from blood IL-9–producing T cells, and hence experiments using differentiated naive-blood T cells might not completely reflect the situation in the human skin.

To propagate and to determine the local regulation and biology of T cells with a focus on IL-9–producing T cells, we cultured *ex vivo* skin in a medium containing T_h_9-differentiating cytokines IL-2, IL-4, and TGF-β (T_h_9-PC). As previously reported in Clark *et al.* (5), emigrated cells were almost exclusively memory T cells with a predominance of CD4 T cells (40). We found a significantly lower expression of CCR4 on cells emigrated from IL-2 and IL-15 conditions. Our data are in contrast with results of a previous report demonstrating higher CCR4 expression after IL-15 stimulation of blood T cells (51). These conflicting results might be explained because of different stimulation periods and differing cell origin. On the contrary, a CD56^+^ cell population was found in SC, possibly explained by the effects of IL-15, a well-known factor involved in NK cell development (52). In T_h_9-PC, we observed a significantly lower frequency of CD69-expressing T cells and found that this effect was mediated *via* IL-4 and TGF-β. In line with our observation are previous results showing inhibition of IL-1β–induced CD69 expression on human naive T cells by TGF-β (53). *In vivo*, CD69 knockout mice showed a marked reduction of skin-resident T cells, highlighting the importance of CD69 in skin retention (6). Furthermore, CD69 suppressive activity of IL-4 was demonstrated in NK cells and CD3^+^CD4^−^CD8^−^ T cells (54). On the contrary, T cells in T_h_9-PC showed increased CD103 expression, even after loss of skin tissue contact, which could be ascribed to TGF-β function as previously demonstrated in CD8^+^ T cells (6, 55).

When addressing the stability of skin T cells, we found in reseeding experiments that IL-9 production by skin T cells seems to be dependent on the cytokine milieu. Deprivation of T_h_9-PC upon 4 wk of culture and reseeding of T cells under SC conditions leads to a significant decrease of IL-9–producing T cells, suggesting a plastic phenotype. In contrast, when skin T cells cultured initially under T_h_9-PC were reseeded under the same conditions, the IL-9–producing T-cell frequency steadily increased. Analysis of the cytokine profile of single cells revealed that the majority of IL-9–producing T cells did not coexpress any of the other T_h_ cytokines and hence constitute a distinct T-cell subset.

IL-6 is an important survival factor for T cells, a growth factor for B cells (56), boosts T cell activation besides IL-2, promotes T_h_2 differentiation, and is a critical cytokine produced by antigen presenting cells (56–59). Additionally, IL-6 improves T_h_9 cell differentiation when added to T_h_9 polarizing conditions (60). In Fig. 4*A*, we showed that more T cells secreting IFN-γ (T_h_1) (61) and IL-13 (T_h_2) exist in cultures with SC compared with T_h_9-PC. Because T_h_1 cells are the main producers of TNF-α, and T_h_2 cells are an important source of IL-6 among other T_h_2-related cytokines, it is conceivable that they are responsible for the higher cytokine levels in the SC group compared with the T_h_9-PC group. In contrast, we found more IL-17^+^ T cells in the T_h_9-PC group than in the SC group, which might be due to the presence of the T_h_17-promoting cytokines TGF-β (added experimentally) and IL-6 (produced by skin cells).

When comparing supernatants derived from T-cell and skin explant cultures, differences with regard to IL-4, IL-6, and TNF-α cytokine levels were observed. Of note, supernatants as shown in Supplemental Fig. S1*F* were harvested after 4 wk of skin culture, in which, other than T cells, fibroblasts present in the cultures may also release cytokines (62), whereas supernatants in Fig. 4*B* were obtained from purified and stimulated T cells derived from 4 wk skin explant cultures only. Indeed, we consistently observed more fibroblasts in wells of the T_h_9-PC group compared with the SC group (unpublished results). Because the numbers of T cells were not determined in skin-cell explant cultures but rather in stimulated T-cell cultures only, different T-cell densities may also explain the different cytokine levels.

RNA-seq and subsequent functional annotation analysis found mainly gene clusters associated with major histocompatibility complex class II–antigen presentation and cell-cell adhesion in T_h_9-PC as well as endoplasmic reticulum-to-Golgi vesicle–mediated transport and type I IFN signaling pathway in SC compared with FI skin T cells. HLA-DR has been described as a mid-to-late T-cell activation protein, and its enhanced mRNA expression in T_h_9-PC indicates a higher activation status than in FI T cells. IL-4 is important for HLA-DR up-regulation (63, 64). Contamination with other cell types can be ruled out because typically ∼99% of T_h_9-PC T cells are CD3^+^. Interestingly, genes of the type I IFN pathway were up-regulated in SC, which might imply presence of α/β IFN.

We found that the top hits *CXCL8* and *CXCL13* identified by RNA-seq analysis, which are higher expressed by T_h_9-PC compared with SC T cells, positively affected neutrophil survival. Skin T cells including IL-9-producing T cells might maintain neutrophils at a site of inflammation *via* this mechanism. Interestingly, CXCL13 also increased neutrophil survival, although this chemokine was mainly described as a B-cell attraction factor (65).

In summary, we present a comprehensive report about the alteration of the skin-derived T-cell phenotype and functional properties in response to the cytokine environment. This might open new avenues for future studies to elucidate a possible therapeutic influence on T-cell–associated skin diseases.

## Supplementary Material

This article includes supplemental data. Please visit *http://www.fasebj.org* to obtain this information.

## Abbreviations

7-AAD: 7-aminoactinomycin D
APC: allophycocyanin
CLA: cutaneous lymphocyte-associated antigen
CSC: cell-stimulation cocktail
FI: freshly isolated
HLA: human leukocyte antigen
IRF4: IFN regulatory factor 4
MPO: myeloperoxidase
NK: natural killer
PBMC: peripheral blood mononuclear cell
RNA-seq: RNA sequencing
RPMI: Roswell Park Memorial Institute
SC: standard condition
STAT: signal transducer and activator of transcription
T_h_: T helper
T_h_9-PC: T_h_9-promoting condition
T_reg_: regulatory T

## References

[1] Streilein J. W. (1983) Skin-associated lymphoid tissues (SALT): origins and functions. J. Invest. Dermatol. 80 (Suppl), 12s–16s 6602189 10.1111/1523-1747.ep12536743

[2] Bos J. D., Kapsenberg M. L. (1986) The skin immune system its cellular constituents and their interactions. Immunol. Today 7, 235–240 25290406 10.1016/0167-5699(86)90111-8

[3] Netea M. G., Brown G. D., Kullberg B. J., Gow N. A. R. (2008) An integrated model of the recognition of Candida albicans by the innate immune system. Nat. Rev. Microbiol. 6, 67–78 18079743 10.1038/nrmicro1815

[4] Nestle F. O., Di Meglio P., Qin J. Z., Nickoloff B. J. (2009) Skin immune sentinels in health and disease. Nat. Rev. Immunol. 9, 679–691 19763149 10.1038/nri2622 PMC2947825

[5] Clark R. A., Chong B., Mirchandani N., Brinster N. K., Yamanaka K., Dowgiert R. K., Kupper T. S. (2006) The vast majority of CLA+ T cells are resident in normal skin. J. Immunol. 176, 4431–4439 16547281 10.4049/jimmunol.176.7.4431

[6] Mackay L. K., Rahimpour A., Ma J. Z., Collins N., Stock A. T., Hafon M.-L., Vega-Ramos J., Lauzurica P., Mueller S. N., Stefanovic T., Tscharke D. C., Heath W. R., Inouye M., Carbone F. R., Gebhardt T. (2013) The developmental pathway for CD103(+)CD8+ tissue-resident memory T cells of skin. Nat. Immunol. 14, 1294–1301 24162776 10.1038/ni.2744

[7] Mackay L. K., Braun A., Macleod B. L., Collins N., Tebartz C., Bedoui S., Carbone F. R., Gebhardt T. (2015) Cutting edge: CD69 interference with sphingosine-1-phosphate receptor function regulates peripheral T cell retention. J. Immunol. 194, 2059–2063 25624457 10.4049/jimmunol.1402256

[8] Watanabe R., Gehad A., Yang C., Scott L. L., Teague J. E., Schlapbach C., Elco C. P., Huang V., Matos T. R., Kupper T. S., Clark R. A. (2015) Human skin is protected by four functionally and phenotypically discrete populations of resident and recirculating memory T cells. Sci. Transl. Med. 7, 279ra39 10.1126/scitranslmed.3010302 PMC4425193 25787765

[9] Cepek K. L., Shaw S. K., Parker C. M., Russell G. J., Morrow J. S., Rimm D. L., Brenner M. B. (1994) Adhesion between epithelial cells and T lymphocytes mediated by E-cadherin and the α E β 7 integrin. Nature 372, 190–193 7969453 10.1038/372190a0

[10] Gebhardt T., Wakim L. M., Eidsmo L., Reading P. C., Heath W. R., Carbone F. R. (2009) Memory T cells in nonlymphoid tissue that provide enhanced local immunity during infection with herpes simplex virus. Nat. Immunol. 10, 524–530 19305395 10.1038/ni.1718

[11] Berg E. L., Yoshino T., Rott L. S., Robinson M. K., Warnock R. A., Kishimoto T. K., Picker L. J., Butcher E. C. (1991) The cutaneous lymphocyte antigen is a skin lymphocyte homing receptor for the vascular lectin endothelial cell-leukocyte adhesion molecule 1. J. Exp. Med. 174, 1461–1466 1720810 10.1084/jem.174.6.1461 PMC2119052

[12] Kieffer J. D., Fuhlbrigge R. C., Armerding D., Robert C., Ferenczi K., Camphausen R. T., Kupper T. S. (2001) Neutrophils, monocytes, and dendritic cells express the same specialized form of PSGL-1 as do skin-homing memory T cells: cutaneous lymphocyte antigen. Biochem. Biophys. Res. Commun. 285, 577–587 11453631 10.1006/bbrc.2001.5230

[13] Yoshie O., Matsushima K. (2015) CCR4 and its ligands: from bench to bedside. Int. Immunol. 27, 11–20 25087232 10.1093/intimm/dxu079

[14] Schlapbach C., Gehad A., Yang C., Watanabe R., Guenova E., Teague J. E., Campbell L., Yawalkar N., Kupper T. S., Clark R. A. (2014) Human TH9 cells are skin-tropic and have autocrine and paracrine proinflammatory capacity. Sci. Transl. Med. 6, 219ra8 10.1126/scitranslmed.3007828 PMC4102325 24431112

[15] Sanchez Rodriguez R., Pauli M. L., Neuhaus I. M., Yu S. S., Arron S. T., Harris H. W., Yang S. H., Anthony B. A., Sverdrup F. M., Krow-Lucal E., MacKenzie T. C., Johnson D. S., Meyer E. H., Löhr A., Hsu A., Koo J., Liao W., Gupta R., Debbaneh M. G., Butler D., Huynh M., Levin E. C., Leon A., Hoffman W. Y., McGrath M. H., Alvarado M. D., Ludwig C. H., Truong H. A., Maurano M. M., Gratz I. K., Abbas A. K., Rosenblum M. D. (2014) Memory regulatory T cells reside in human skin. J. Clin. Invest. 124, 1027–1036 24509084 10.1172/JCI72932 PMC3934172

[16] Clark R. A., Kupper T. S. (2007) IL-15 and dermal fibroblasts induce proliferation of natural regulatory T cells isolated from human skin. Blood 109, 194–202 16968902 10.1182/blood-2006-02-002873 PMC1785078

[17] Purwar R., Schlapbach C., Xiao S., Kang H. S., Elyaman W., Jiang X., Jetten A. M., Khoury S. J., Fuhlbrigge R. C., Kuchroo V. K., Clark R. A., Kupper T. S. (2012) Robust tumor immunity to melanoma mediated by interleukin-9-producing T cells. Nat. Med. 18, 1248–1253 22772464 10.1038/nm.2856 PMC3518666

[18] Vieyra-Garcia P. A., Wei T., Naym D. G., Fredholm S., Fink-Puches R., Cerroni L., Odum N., O’Malley J. T., Gniadecki R., Wolf P. (2016) STAT3/5-dependent IL9 overexpression contributes to neoplastic cell survival in mycosis fungoides. Clin. Cancer Res. 22, 3328–3339 26851186 10.1158/1078-0432.CCR-15-1784 PMC4967550

[19] Schmitt E., Germann T., Goedert S., Hoehn P., Huels C., Koelsch S., Kühn R., Müller W., Palm N., Rüde E. (1994) IL-9 production of naive CD4+ T cells depends on IL-2, is synergistically enhanced by a combination of TGF-beta and IL-4, and is inhibited by IFN-gamma. J. Immunol. 153, 3989–3996 7930607

[20] Kaplan M. H. (2017) The transcription factor network in Th9 cells. Semin. Immunopathol. 39, 11–20 27837254 10.1007/s00281-016-0600-2 PMC5225158

[21] Clark R. A., Schlapbach C. (2017) T_H_9 cells in skin disorders. Semin. Immunopathol. 39, 47–54 27896634 10.1007/s00281-016-0607-8 PMC5471354

[22] Partridge M., Green M. R., Langdon J. D., Feldmann M. (1989) Production of TGF-α and TGF-β by cultured keratinocytes, skin and oral squamous cell carcinomas--potential autocrine regulation of normal and malignant epithelial cell proliferation. Br. J. Cancer 60, 542–548 2478181 10.1038/bjc.1989.310 PMC2247118

[23] Schuster C., Vaculik C., Fiala C., Meindl S., Brandt O., Imhof M., Stingl G., Eppel W., Elbe-Bürger A. (2009) HLA-DR^+^ leukocytes acquire CD1 antigens in embryonic and fetal human skin and contain functional antigen-presenting cells. J. Exp. Med. 206, 169–181 19139172 10.1084/jem.20081747 PMC2626673

[24] Van der Graaf C. A. A., Netea M. G., Franke B., Girardin S. E., van der Meer J. W. M., Kullberg B. J. (2006) Nucleotide oligomerization domain 2 (Nod2) is not involved in the pattern recognition of Candida albicans. Clin. Vaccine Immunol. 13, 423–425 16522788 10.1128/CVI.13.3.423-425.2006 PMC1391959

[25] Zhu J. (2015) T helper 2 (Th2) cell differentiation, type 2 innate lymphoid cell (ILC2) development and regulation of interleukin-4 (IL-4) and IL-13 production. Cytokine 75, 14–24 26044597 10.1016/j.cyto.2015.05.010 PMC4532589

[26] Nelson B. H. (2004) IL-2, regulatory T cells, and tolerance. J. Immunol. 172, 3983–3988 15034008 10.4049/jimmunol.172.7.3983

[27] Michalak-Stoma A., Pietrzak A., Szepietowski J. C., Zalewska-Janowska A., Paszkowski T., Chodorowska G. (2011) Cytokine network in psoriasis revisited. Eur. Cytokine Netw. 22, 160–168 22236965 10.1684/ecn.2011.0294

[28] Liao W., Schones D. E., Oh J., Cui Y., Cui K., Roh T. Y., Zhao K., Leonard W. J. (2008) Priming for T helper type 2 differentiation by interleukin 2-mediated induction of interleukin 4 receptor α-chain expression. Nat. Immunol. 9, 1288–1296 18820682 10.1038/ni.1656 PMC2762127

[29] Chang H.-C., Sehra S., Goswami R., Yao W., Yu Q., Stritesky G. L., Jabeen R., McKinley C., Ahyi A.-N., Han L., Nguyen E. T., Robertson M. J., Perumal N. B., Tepper R. S., Nutt S. L., Kaplan M. H. (2010) The transcription factor PU.1 is required for the development of IL-9-producing T cells and allergic inflammation. Nat. Immunol. 11, 527–534 20431622 10.1038/ni.1867 PMC3136246

[30] Goswami R., Jabeen R., Yagi R., Pham D., Zhu J., Goenka S., Kaplan M. H. (2012) STAT6-dependent regulation of Th9 development. J. Immunol. 188, 968–975 22180613 10.4049/jimmunol.1102840 PMC3262957

[31] Salimi M., Subramaniam S., Selvakumar T., Wang X., Zemenides S., Johnson D., Ogg G. (2016) Enhanced isolation of lymphoid cells from human skin. Clin. Exp. Dermatol. 41, 552–556 26805629 10.1111/ced.12802 PMC4981906

[32] Gschwandtner M., Kienzl P., Tajpara P., Schuster C., Stipek G., Buchberger M., Mildner M., Mairhofer M., Eppel W., Vierhapper M., Pammer J., Koller R., Elbe-Bürger A., Tschachler E. (2018) The reticulum-associated protein RTN1A specifically identifies human dendritic cells. J. Invest. Dermatol. 138, 1318–1327 29369773 10.1016/j.jid.2018.01.002

[33] Polak D., Hafner C., Briza P., Kitzmüller C., Elbe-Bürger A., Samadi N., Gschwandtner M., Pfützner W., Zlabinger G. J., Jahn-Schmid B., Bohle B. (2018) A novel role for neutrophils in IgE-mediated allergy: evidence for antigen presentation in late-phase reactions. [E-pub ahead of print] J. Allergy Clin. Immunol. 10.1016/j.jaci.2018.06.005 10.1016/j.jaci.2018.06.005 PMC6986898 29920351

[34] Stassen M., Arnold M., Hültner L., Müller C., Neudörfl C., Reineke T., Schmitt E. (2000) Murine bone marrow-derived mast cells as potent producers of IL-9: costimulatory function of IL-10 and kit ligand in the presence of IL-1. J. Immunol. 164, 5549–5555 10820228 10.4049/jimmunol.164.11.5549

[35] Stassen M., Müller C., Arnold M., Hültner L., Klein-Hessling S., Neudörfl C., Reineke T., Serfling E., Schmitt E. (2001) IL-9 and IL-13 production by activated mast cells is strongly enhanced in the presence of lipopolysaccharide: NF-kappa B is decisively involved in the expression of IL-9. J. Immunol. 166, 4391–4398 11254693 10.4049/jimmunol.166.7.4391

[36] Stassen M., Klein M., Becker M., Bopp T., Neudörfl C., Richter C., Heib V., Klein-Hessling S., Serfling E., Schild H., Schmitt E. (2007) p38 MAP kinase drives the expression of mast cell-derived IL-9 via activation of the transcription factor GATA-1. Mol. Immunol. 44, 926–933 16650898 10.1016/j.molimm.2006.03.019

[37] Licona-Limón P., Henao-Mejia J., Temann A. U., Gagliani N., Licona-Limón I., Ishigame H., Hao L., Herbert D. R., Flavell R. A. (2013) Th9 cells drive host immunity against gastrointestinal worm infection. Immunity 39, 744–757 24138883 10.1016/j.immuni.2013.07.020 PMC3881610

[38] Wilhelm C., Hirota K., Stieglitz B., Van Snick J., Tolaini M., Lahl K., Sparwasser T., Helmby H., Stockinger B. (2011) An IL-9 fate reporter demonstrates the induction of an innate IL-9 response in lung inflammation. Nat. Immunol. 12, 1071–1077 21983833 10.1038/ni.2133 PMC3198843

[39] Guo S., Li Z.-Z., Jiang D.-S., Lu Y. Y., Liu Y., Gao L., Zhang S.-M., Lei H., Zhu L.-H., Zhang X.-D., Liu D.-P., Li H. (2014) IRF4 is a novel mediator for neuronal survival in ischaemic stroke. Cell Death Differ. 21, 888–903 24510125 10.1038/cdd.2014.9 PMC4013523

[40] Clark R. A., Chong B. F., Mirchandani N., Yamanaka K., Murphy G. F., Dowgiert R. K., Kupper T. S. (2006) A novel method for the isolation of skin resident T cells from normal and diseased human skin. J. Invest. Dermatol. 126, 1059–1070 16484986 10.1038/sj.jid.5700199

[41] Cheng G. Z., Park S., Shu S., He L., Kong W., Zhang W., Yuan Z., Wang L.-H., Cheng J. Q. (2008) Advances of AKT pathway in human oncogenesis and as a target for anti-cancer drug discovery. Curr. Cancer Drug Targets 8, 2–6 18288938

[42] Aratani Y. (2018) Myeloperoxidase: its role for host defense, inflammation, and neutrophil function. Arch. Biochem. Biophys. 640, 47–52 29336940 10.1016/j.abb.2018.01.004

[43] El Kebir D., Filep J. G. (2013) Modulation of neutrophil apoptosis and the resolution of inflammation through β2 integrins. Front. Immunol. 4, 60 23508943 10.3389/fimmu.2013.00060 PMC3589696

[44] Hirahara K., Poholek A., Vahedi G., Laurence A., Kanno Y., Milner J. D., O’Shea J. J. (2013) Mechanisms underlying helper T-cell plasticity: implications for immune-mediated disease. J. Allergy Clin. Immunol. 131, 1276–1287 23622118 10.1016/j.jaci.2013.03.015 PMC3677748

[45] Perlroth J., Choi B., Spellberg B. (2007) Nosocomial fungal infections: epidemiology, diagnosis, and treatment. Med. Mycol. 45, 321–346 17510856 10.1080/13693780701218689

[46] Erwig L. P., Gow N. A. R. (2016) Interactions of fungal pathogens with phagocytes. Nat. Rev. Microbiol. 14, 163–176 26853116 10.1038/nrmicro.2015.21

[47] Gerlach K., Hwang Y., Nikolaev A., Atreya R., Dornhoff H., Steiner S., Lehr H. A., Wirtz S., Vieth M., Waisman A., Rosenbauer F., McKenzie A. N. J., Weigmann B., Neurath M. F. (2014) TH9 cells that express the transcription factor PU.1 drive T cell-mediated colitis via IL-9 receptor signaling in intestinal epithelial cells. Nat. Immunol. 15, 676–686 24908389 10.1038/ni.2920

[48] Jabeen R., Kaplan M. H. (2012) The symphony of the ninth: the development and function of Th9 cells. Curr. Opin. Immunol. 24, 303–307 22365614 10.1016/j.coi.2012.02.001 PMC3368035

[49] Liu J., Harberts E., Tammaro A., Girardi N., Filler R. B., Fishelevich R., Temann A., Licona-Limón P., Girardi M., Flavell R. A., Gaspari A. A. (2014) IL-9 regulates allergen-specific Th1 responses in allergic contact dermatitis. J. Invest. Dermatol. 134, 1903–1911 24487305 10.1038/jid.2014.61 PMC4303591

[50] Humblin E., Thibaudin M., Chalmin F., Derangère V., Limagne E., Richard C., Flavell R. A., Chevrier S., Ladoire S., Berger H., Boidot R., Apetoh L., Végran F., Ghiringhelli F. (2017) IRF8-dependent molecular complexes control the Th9 transcriptional program. Nat. Commun. 8, 2085 29233972 10.1038/s41467-017-01070-w PMC5727025

[51] Norii M., Yamamura M., Iwahashi M., Ueno A., Yamana J., Makino H. (2006) Selective recruitment of CXCR3+ and CCR5+ CCR4+ T cells into synovial tissue in patients with rheumatoid arthritis. Acta Med. Okayama 60, 149–157 16838043 10.18926/AMO/30745

[52] Cooper M. A., Fehniger T. A., Turner S. C., Chen K. S., Ghaheri B. A., Ghayur T., Carson W. E., Caligiuri M. A. (2001) Human natural killer cells: a unique innate immunoregulatory role for the CD56(bright) subset. Blood 97, 3146–3151 11342442 10.1182/blood.v97.10.3146

[53] Hebel K., Rudolph M., Kosak B., Chang H.-D., Butzmann J., Brunner-Weinzierl M. C. (2011) IL-1β and TGF-β act antagonistically in induction and differentially in propagation of human proinflammatory precursor CD4+ T cells. J. Immunol. 187, 5627–5635 22048775 10.4049/jimmunol.1003998

[54] Gerosa F., Tommasi M., Carra G., Gandini G., Tridente G., Benati C. (1992) Different sensitivity to interleukin 4 of interleukin 2- and interferon α-induced CD69 antigen expression in human resting NK cells and CD3+, CD4-, CD8- lymphocytes. Cell. Immunol. 141, 342–351 1533569 10.1016/0008-8749(92)90153-g

[55] Wang D., Yuan R., Feng Y., El-Asady R., Farber D. L., Gress R. E., Lucas P. J., Hadley G. A. (2004) Regulation of CD103 expression by CD8+ T cells responding to renal allografts. J. Immunol. 172, 214–221 14688328 10.4049/jimmunol.172.1.214

[56] Hirano T., Yasukawa K., Harada H., Taga T., Watanabe Y., Matsuda T., Kashiwamura S., Nakajima K., Koyama K., Iwamatsu A., Tsunasawa S., Sakiyama F., Matsui H., Takahara Y., Taniguchi T., Kishimoto T. (1986) Complementary DNA for a novel human interleukin (BSF-2) that induces B lymphocytes to produce immunoglobulin. Nature 324, 73–76 3491322 10.1038/324073a0

[57] Teague T. K., Marrack P., Kappler J. W., Vella A. T. (1997) IL-6 rescues resting mouse T cells from apoptosis. J. Immunol. 158, 5791–5796 9190930

[58] Rincón M., Anguita J., Nakamura T., Fikrig E., Flavell R. A. (1997) Interleukin (IL)-6 directs the differentiation of IL-4-producing CD4+ T cells. J. Exp. Med. 185, 461–469 9053446 10.1084/jem.185.3.461 PMC2196041

[59] Dienz O., Rincon M. (2009) The effects of IL-6 on CD4 T cell responses. Clin. Immunol. 130, 27–33 18845487 10.1016/j.clim.2008.08.018 PMC2660866

[60] Wong M. T., Ye J. J., Alonso M. N., Landrigan A., Cheung R. K., Engleman E., Utz P. J. (2010) Regulation of human Th9 differentiation by type I interferons and IL-21. Immunol. Cell Biol. 88, 624–631 20421880 10.1038/icb.2010.53 PMC3090036

[61] Romagnani S. (1999) Th1/Th2 cells. Inflamm. Bowel Dis. 5, 285–294 10579123 10.1097/00054725-199911000-00009

[62] Kamimura D., Ishihara K., Hirano T. (2003) IL-6 signal transduction and its physiological roles: the signal orchestration model. Rev. Physiol. Biochem. Pharmacol. 149, 1–38 12687404 10.1007/s10254-003-0012-2

[63] Salgado F. J., Lojo J., Fernández-Alonso C. M., Viñuela J., Cordero O. J., Nogueira M. (2002) Interleukin-dependent modulation of HLA-DR expression on CD4 and CD8 activated T cells. Immunol. Cell Biol. 80, 138–147 11940114 10.1046/j.1440-1711.2002.01055.x

[64] Bang K., Lund M., Mogensen S. C., Thestrup-Pedersen K. (2005) In vitro culture of skin-homing T lymphocytes from inflammatory skin diseases. Exp. Dermatol. 14, 391–397 15854134 10.1111/j.0906-6705.2005.00294.x

[65] Takagi R., Higashi T., Hashimoto K., Nakano K., Mizuno Y., Okazaki Y., Matsushita S. (2008) B cell chemoattractant CXCL13 is preferentially expressed by human Th17 cell clones. J. Immunol. 181, 186–189 18566383 10.4049/jimmunol.181.1.186

