# Supplementary material for: The cytokine environment influence on human skin–derived T cells
Source: FASEB J. 2019 Feb 26;33(5):6514–25. doi: 10.1096/fj.201801416R (PMC6463918; doi:10.1096/fj.201801416R)
Supplement: Supplementary file 1 [file fj.201801416R.sd1.docx]

# SUPPLEMENTARY MATERIALS AND METHODS

## Immunofluorescence staining

T cells (2x10^4^/spot) were placed on adhesion slides (Marienfeld, Germany). Adhesion slides and cryosections (5µm) of healthy human skin were fixed in acetone for 10 min, air-dried and rehydrated in PBS. Staining was performed with antibodies (Supplementary Table 1) over night at 4°C. On the next day, slides were washed in PBS and secondary antibodies (see Materials and Methods section) were added for 1 h at room temperature. Nuclei were counterstained with 4’,6-Diamidine-2’-phenylindole dihydrochloride (DAPI; Sigma Aldrich, USA). Background fluorescence was adjusted to matched isotype control antibody staining.

## Flow cytometry

After dead cell exclusion using fixable viability dye eFluor 450 (Thermo Fisher Scientific, USA), cells were incubated with antibodies (Supplementary Table 1) for 20 min at 4°C. eBioscience IC Fixation Buffer or for intranuclear staining Foxp3 / Transcription Factor Staining Buffer Set (both Thermo Fisher, USA) were used for cell fixation. Intracellular staining (Supplementary Table 1) was performed at room temperature for 20 min. Matched isotype control antibodies were used to determine staining above autofluorescence. Cellular debris and doublets were excluded using forward scatter area and side scatter area gating and forward scatter area and forward scatter width gating, respectively. For surface protein staining only, single cell suspensions were stained with the indicated antibodies and dead cells were excluded using DAPI.

## Western Blot

Samples and Precision Plus Protein WesternC Standard (Bio-Rad, USA) were loaded on Any kD Criterion TGX Precast Gels (Bio-Rad, USA) and proteins were transferred onto nitrocellulose membranes using the Trans-Blot Turbo system (Bio-Rad, USA). Equal protein loading was visualized with Ponceau S staining (Merck, Germany). After washing, the membranes were incubated with the indicated antibodies (Supplementary Table 1) at 4°C over night. GAPDH was included as reference protein. The next day the membranes were washed and incubated for 1 h with HRP-coupled sheep αrabbit secondary antibody (Bio-Rad, USA). SuperSignal West Dura Extended Duration Substrate (Thermo Fisher Scientific, USA) was added and chemiluminescence was detected on a Molecular Imager ChemiDoc XRS+ with ImageLab v 4.1 software (Bio-Rad, USA).

## Transcriptome analyses

Quality control of RNA samples was performed using RNA 6000 Nano Kit on a 2100 Bioanalyzer (Agilent, USA). Libraries were QC-checked on a Bioanalyzer 2100 (Agilent, USA) using a High Sensitivity DNA Kit for correct insert size and quantitated using Qubit dsDNA HS Assay (Invitrogen, USA). Pooled libraries hand an average length of 330-360bp and were sequenced on a NextSeq500 instrument (Illumina, USA) in 2 x 75 bp sequencing mode. Trimmomatic v0.36 (Bolger et al., Bioinformatics. 30 (15): 2114–20, 2014) was used to remove adapter sequences of reads, quality check was performed using FastQC v0.11.7. For gene-level expression the HISAT2 v2.1 (Kim et al., Nature Methods. 12 (4): 357–60, 2015), StringTie v1.3.4c (Pertea et al., Nature Biotechnology. 33 (3): 290–5, 2015) and Ballgown v2.10.0 (Frazee et al., Nature Biotechnology. 33 (3): 243–6, 2015) tools were applied essentially as described (Pertea et al., Nature protocols. 11 (9): 1650–67, 2016). Further data processing and visualization were conducted in R v3.4.3. Functional annotation analysis was conducted using the DAVID tool (Huang et al., Nature Protocols. 4 (1): 44–57, 2009). The dataset was uploaded to the Gene Expression Omnibus (GEO) database and can be accessed via number GSE123320.
