# Supplementary material for: The cytokine environment influence on human skin–derived T cells
Source: FASEB J. 2019 Feb 26;33(5):6514–25. doi: 10.1096/fj.201801416R (PMC6463918; doi:10.1096/fj.201801416R)
Supplement: Supplementary file 2 [file fj.201801416R.sd2.docx]

# SUPPLEMENTARY Figure Legends

**Supplementary Figure 1. Characterization of TH9 cells in situ and of cells collected from skin explant cultures.** (**a**) Two-color immunofluorescence staining of CD3 and IL-9 in cryosections of human skin. White arrow marks an IL-9^+^ T cell (insert) within the lower dermal compartment. The dotted line shows the dermal-epidermal junction. Scale bar = 50 µm. (**b**) Growth curve of emigrated cells at the indicated culture conditions (n=3). (**c-e**) Representative Ki-67, CD56 and FoxP3 staining (left) and respective quantification dot plots (right; n=4-9) analyzed after 4 weeks of culture. Quadrants were set according to isotype-matched control staining. (**f**) Bar graph illustrating cytokine levels of skin explant culture supernatants after 4 weeks of culture (n=7). Data are expressed as mean ± standard error of the mean (SEM) and were analyzed using Wilcoxon matched pairs signed-rank test (**c, d and f**) or Mann-Whitney U test (**e**). * P ≤ 0.05. nd, below detection limit.

**Supplementary Figure 2. Transcriptional profile and functional annotation analysis.** (**a**) MA-plot of differentially regulated genes between TH9-PC (PC) and SC. Significantly more than 4-fold differentially regulated genes are marked with gene-symbol. (**b**) Dot plot of HLA-DRB1 and CD3 on T cells from TH9-PC and SC determined using flow cytometry. One representative donor is shown. (**c**) Bar diagram shows IL-9 expression (FPKM values) in TH9-PC and SC as determined by RNA-seq. (**d**) Functional annotation analysis of significantly up- or downregulated genes in the indicated groups using the DAVID tool listing functional annotation clusters.

**Supplementary Figure 3. CXCL8 and CXCL13 increase neutrophil survival and diminish MPO production.** (**a**) Percentage of CCR4^+^ cells among skin T cells (freshly isolated (FI) or harvested after 4 weeks from skin explant cultures under indicated conditions). Only significant differences are indicated (n = 4). (**b**) Bar plot summarizing the frequency of skin homing marker expressing blood-derived T cells upon culture for 48 h in medium alone (ctrl), medium containing 100 ng/ml CXCL8, 100 ng/ml CXCL13 or both (n = 3). (**c**) Quantification of neutrophils and PBMCs migrated through the filter membrane into the bottom well in the presence and absence (ctrl) of CXCL8 and CXCL13, respectively. (**d**) Percentage of living neutrophils as determined by Annexin V / 7-AAD staining and (**e**) Myeloperoxidase (MPO) protein levels normalized to medium only control (ctrl) are shown. Supernatants from 3-day αCD3/αCD28 bead stimulated T cells derived from skin explant cultures after 4 weeks of 6 donors at the indicated conditions or cRPMI (ctrl) was added to neutrophil culture. (**f**) Percentage of living neutrophils as determined by Annexin V / 7-AAD staining at culture conditions as in (**c**). RPMI was used as control (ctrl). Three technical replicates of one donor were measured in (**c**) and (**f**). Bar graph indicates mean and standard error of the mean (SEM) and differences were analyzed using paired Student’s t-test * P < 0.05; ** P < 0.01.
