# Supplementary figures and images for: The cytokine environment influence on human skin–derived T cells
Source: FASEB J. 2019 Feb 26;33(5):6514–25. doi: 10.1096/fj.201801416R (PMC6463918; doi:10.1096/fj.201801416R)

# Supplementary Figure 1

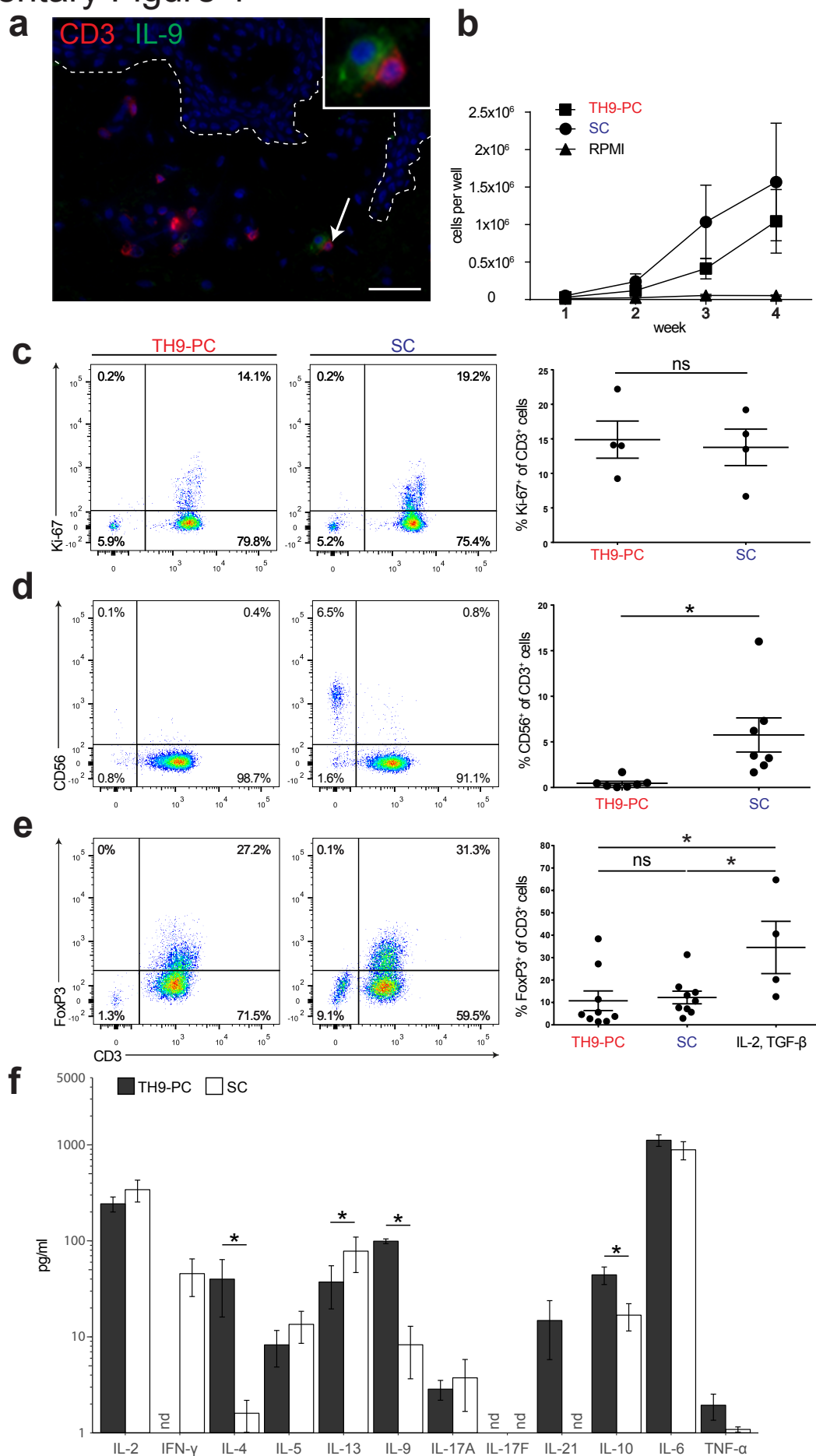

Supplement: Supplementary file 3 [file fj.201801416R.sf1.pdf]

Supplementary Figure 2

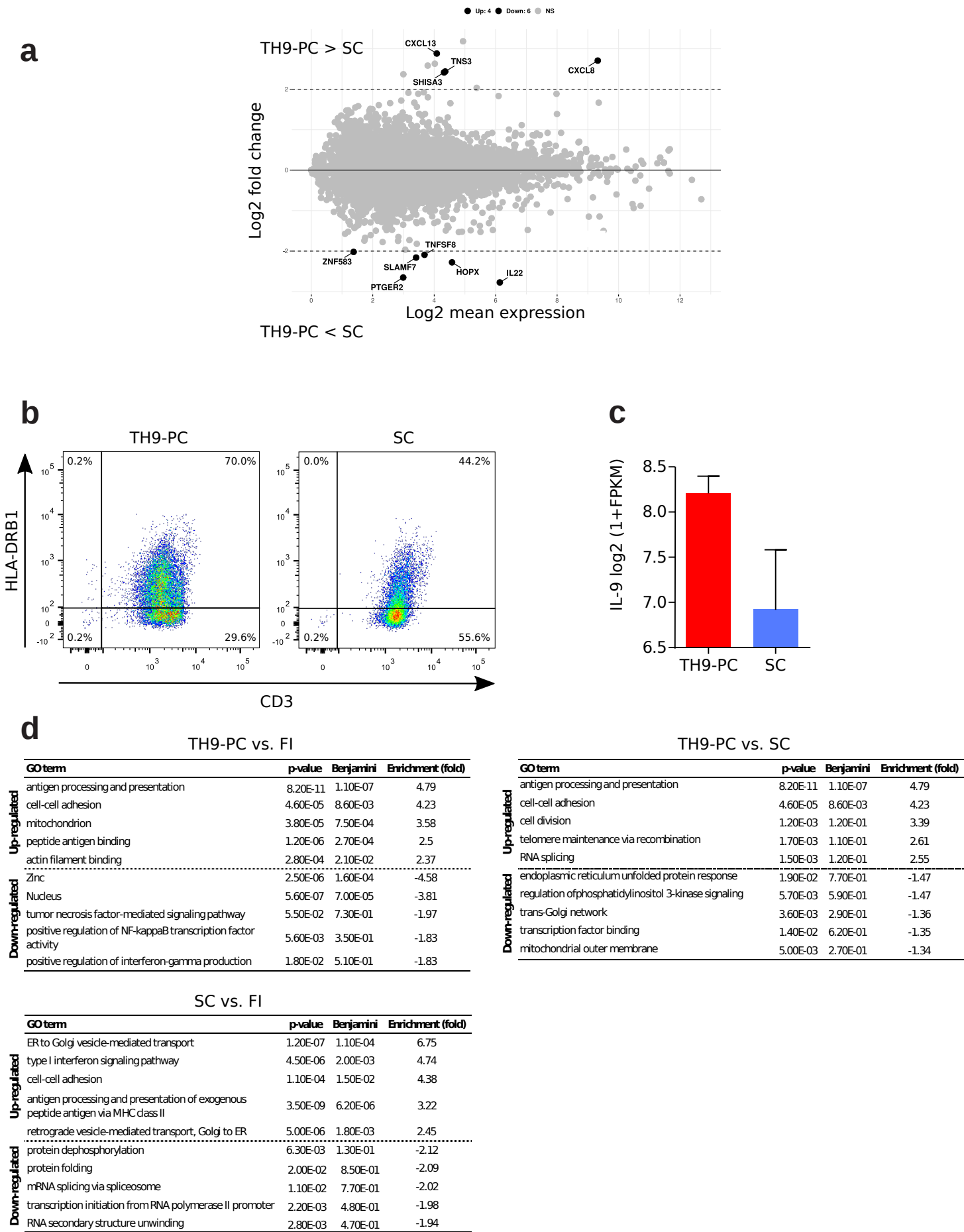

Supplement: Supplementary file 4 [file fj.201801416R.sf2.pdf]

Supplementary Figure 3

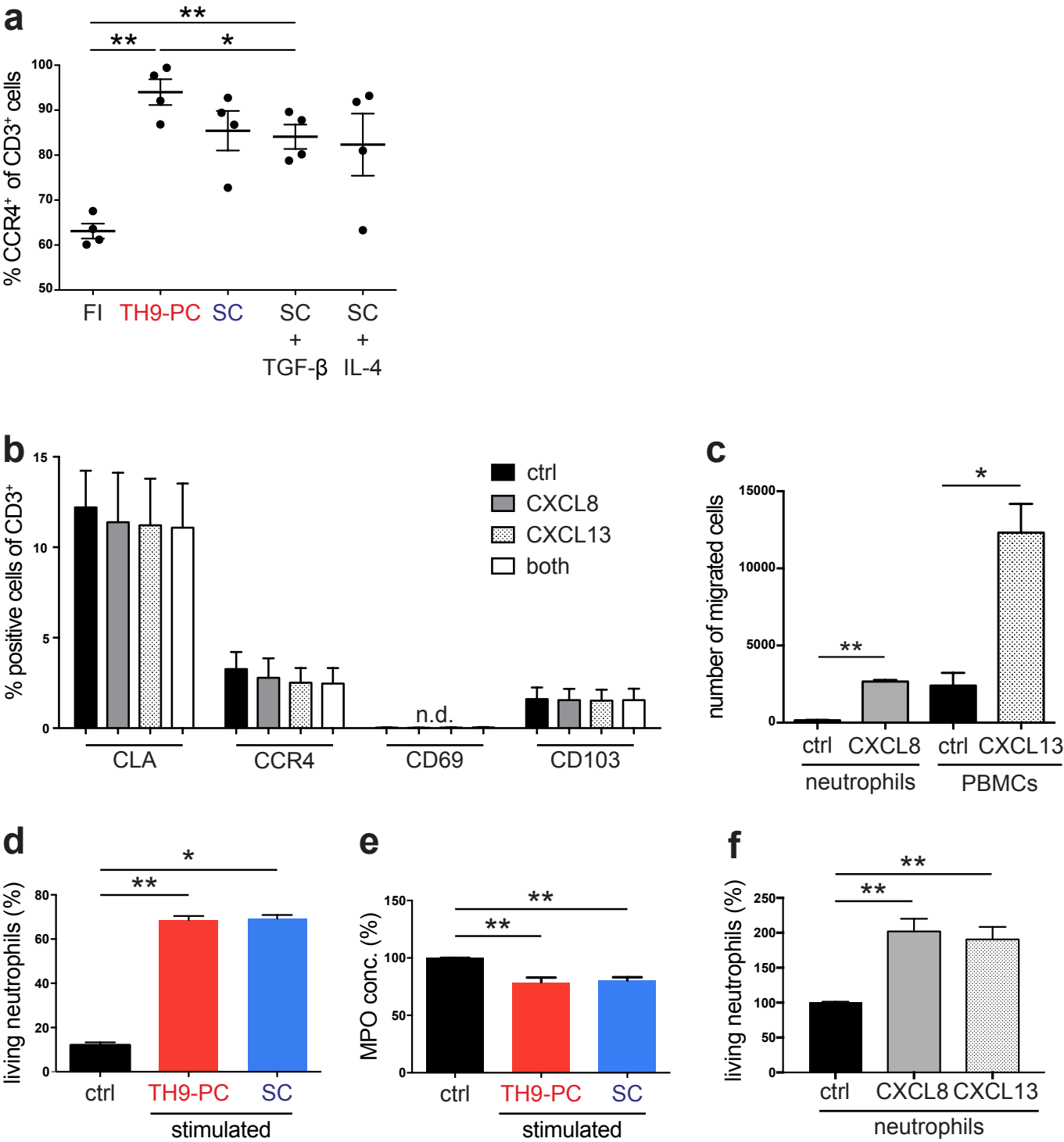

Supplement: Supplementary file 5 [file fj.201801416R.sf3.pdf]
