# Supplementary material for: The cytokine environment influence on human skin–derived T cells
Source: FASEB J. 2019 Feb 26;33(5):6514–25. doi: 10.1096/fj.201801416R (PMC6463918; doi:10.1096/fj.201801416R)
Supplement: Supplementary file 6 [file fj.201801416R.st1.pdf]

Supplementary Table 1. List of antibodies used

Antibody	Conjugate	Clone	Company	Category
CD3	FITC	SK7	BD	flow cytometry (surface)
CD3	PE	SK7	BD	flow cytometry (surface)
CD3	APC	SK7	BD	flow cytometry (surface)
CD4	FITC	SK3	BD	flow cytometry (surface)
CD4	Brilliant Violet 510	SK3	BioLegend	flow cytometry (surface)
CD8	APC	SK1	BD	flow cytometry (surface)
CD8	APC-Bio770	REA734	Miltenyi	flow cytometry (surface)
CD45RA	FITC	T6D11	Miltenyi	flow cytometry (surface)
CD45RO	PE	UCHL1	BD Pharmingen	flow cytometry (surface)
CD56	APC	TULY56	eBioscience (Thermo Fisher)	flow cytometry (surface)
CD69	PE-Cy7	FN50	eBioscience (Thermo Fisher)	flow cytometry (surface)
CD103	PerCP-eFluor710	Ber-ACT8	eBioscience (Thermo Fisher)	flow cytometry (surface)
CCR4	APC	D8SEE	eBioscience (Thermo Fisher)	flow cytometry (surface)
CCR4	APC	205410	R&D Systems	flow cytometry (surface)
CLA	FITC	HECA-452	BioLegend	flow cytometry (surface)
IL-9	PE	MH9A3	BD	flow cytometry (intracellular)
IL-13	FITC	85BRD	eBioscience (Thermo Fisher)	flow cytometry (intracellular)
IL-17A	PerCP-Cy7	BL168	BioLegend	flow cytometry (intracellular)
IFN-γ	PE-Cy7	4S.B3	eBioscience (Thermo Fisher)	flow cytometry (intracellular)
Ki-67	FITC	20Raj1	eBioscience (Thermo Fisher)	flow cytometry (intranuclear)
FoxP3	Alexa647	259D/C7	BD Pharmingen	flow cytometry (intranuclear)
IRF4	unconjugated	polyclonal rabbit	CS&T	immunofluorescence, Western blot
PU.1	unconjugated	9G7	CS&T	immunofluorescence, Western blot
GAPDH	unconjugated	6C5	abcam	Western blot
CD3 abcam	unconjugated	SP7	Abcam	immunofluorescence
IL-9	unconjugated	MH9D1	BioLegend	immunofluorescence
